# Endogenous HIF2A reporter systems for high-throughput functional screening

**DOI:** 10.1038/s41598-018-30499-2

**Published:** 2018-08-13

**Authors:** M. Nazhif Zaini, Saroor A. Patel, Saiful E. Syafruddin, Paulo Rodrigues, Sakari Vanharanta

**Affiliations:** 1 MRC Cancer Unit, University of Cambridge, Hutchison/MRC Research Centre, Box 197, Biomedical Campus, Cambridge, CB2 0XZ United Kingdom; 2 UKM Medical Molecular Biology Institute, Universiti Kebangsaan Malaysia, Jalan Yaa’cob Latiff, Bandar Tun Razak, 56000 Cheras, Kuala Lumpur Malaysia

## Abstract

Tissue-specific transcriptional programs control most biological phenotypes, including disease states such as cancer. However, the molecular details underlying transcriptional specificity is largely unknown, hindering the development of therapeutic approaches. Here, we describe novel experimental reporter systems that allow interrogation of the endogenous expression of HIF2A, a critical driver of renal oncogenesis. Using a focused CRISPR-Cas9 library targeting chromatin regulators, we provide evidence that these reporter systems are compatible with high-throughput screening. Our data also suggests redundancy in the control of cancer type-specific transcriptional traits. Reporter systems such as those described here could facilitate large-scale mechanistic dissection of transcriptional programmes underlying cancer phenotypes, thus paving the way for novel therapeutic approaches.

## Introduction

Genetic loss-of-function screening is a powerful approach for mechanistic dissection of biological phenomena. Functional screens can also be useful for the identification of molecular mediators in various disease states. Given the recent methodological developments, such as improved RNAi and CRISPR-Cas9^1,2^, performing genetic screens *in vitro* has become relatively easy, with the limiting factor now being suitable functional assays that model relevant biology accurately. For example, in the field of cancer biology, *in vitro* assays that measure cell proliferation or survival under various conditions can be powerful^3,4^. However, many relevant cancer phenotypes cannot be easily modelled *in vitro*. On the other hand, even though some *in vivo* genetic screens have been successful^5,6^, large-scale genetic screening *in vivo* remains a challenge. Thus, novel functional assays are needed for high-throughput genetic dissection of clinically relevant cancer dependencies.

An example of a cancer phenotype that displays experimental context-specificity is the role of hypoxia-inducible factor 2 (HIF2A), encoded by the *EPAS1* gene, as a driver of renal tumorigenesis. HIF2A is activated in clear cell renal cell carcinoma (ccRCC) through mutations in the *von Hippel-Lindau tumour suppressor gene* (*VHL*), which under normal conditions targets HIF2A for proteosomal degradation^7^. Under hypoxia and in cancers that have lost *VHL*, a stereotypical genetic event in ccRCC^8^, HIF2A accumulates and drives the expression of multiple target genes, some which mediate renal cancer growth and metastasis^7,9,10^. Despite abundant evidence demonstrating the relevance of the VHL-HIF2A pathway for ccRCC growth *in vivo*, HIF2A inhibition directly or via VHL restoration does not have an obvious growth phenotype in tissue culture systems^7^.

We set out to develop experimental systems that would allow genetic interrogation of the molecular mechanisms underlying tissue-specific HIF2A expression in ccRCC. We established and validated fluorescence-based reporters of endogenous HIF2A expression in ccRCC cell lines. As a proof-of-principle, we conducted a pooled CRISPR-Cas9 screen focusing on chromatin factors, demonstrating the feasibility of using these systems in high-throughput genetic screens. Our screening data suggest that HIF2A expression in ccRCC may be supported by a robust regulatory network, which is insensitive to perturbations in individual chromatin factors. We conclude that genetically engineered systems such as those described here could facilitate large-scale dissection of essential transcriptional programmes underlying cancer phenotypes, with potential implications for the identification of novel targets for cancer therapy development.

## Results

### CRISPR-Cas9-based knock-in of a fluorescent reporter into the endogenous *EPAS1* locus

**Figure 1:**
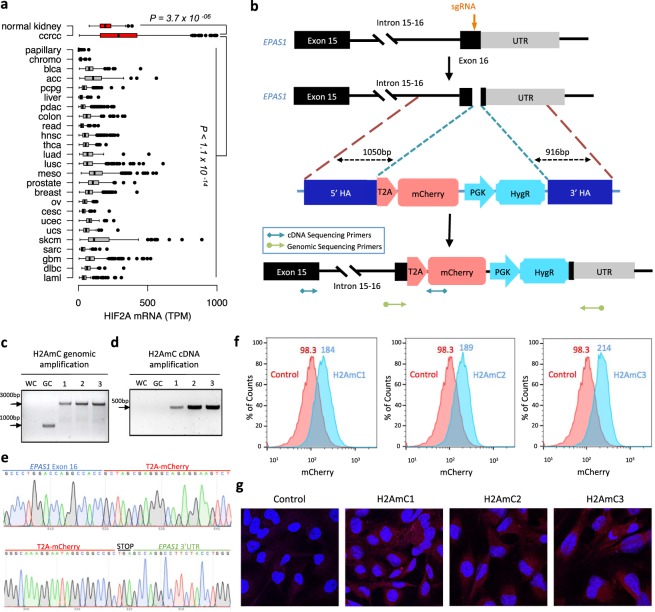
Generation of endogenous HIF2A reporter systems. (**a**) TCGA data analysis of HIF2A mRNA expression in ccRCC compared to different tumour types and normal kidney. P-value by Wilcoxon rank sum test. (**b**) Schematic of CRISPR-Cas9-based knock-in strategy of an mCherry fluorescent gene into the exon 16 of *EPAS1*. Plasmid template consists of an mCherry fluorescent gene with a hygromycin selection marker that are flanked by homology arms (HA). Sequencing primers used for genomic and cDNA amplifications in (**c**) and (**d**), respectively, are shown. (**c**) Genomic amplification of the HIF2A-mCherry integration site in single-cell derived clones. WC, water control. GC, genomic control. (**d**) cDNA amplification of the HIF2A-mCherry integration site. WC, water control. GC, genomic control. (**e**) Sanger sequencing of the HIF2A-mCherry integration sites in H2AmC2 cells. (**f**) FACS analysis of mCherry fluorescence in the three H2AmC clones compared to the parental UOK101 cells. (**g**) mCherry fluorescence in the three H2AmC clones compared to the parental control. DAPI in blue, mCherry in red.

Analysis of RNA-seq data from 26 different tumour types from the TCGA cohort^11^ revealed that HIF2A mRNA was highly expressed in ccRCC when compared to other tumour types (Fig. 1a). HIF2A mRNA expression was also elevated in ccRCC when compared to normal kidney (Fig. 1a). Given that *VHL* mutations are remarkably specific for ccRCC^12,13^, this suggested the possibility that high HIF2A mRNA expression levels in ccRCC may modulate the potential at which *VHL* loss promotes tumorigenesis. To analyse the molecular basis of this transcriptional specificity, we developed a CRISPR-Cas9-based knock-in strategy for the integration of an mCherry fluorescence reporter into the endogenous *EPAS1* locus of ccRCC cells using homology-directed repair (Fig. 1b), an approach that has previously been successfully applied in other biological contexts^14,15^. A T2A self-cleaving peptide was placed between endogenous HIF2A and mCherry, ensuring that HIF2A and mCherry proteins were produced at a stoichiometric ratio^16,17^. After hygromycin selection, surviving cells were sorted into single cell clones based on mCherry fluorescence, followed by genetic analysis of the integration site. Of the cell lines tested, we identified three clones (henceforth referred to as HIF2A-mCherry-single-cell-clone 1–3 or H2AmC1–3), all derived from UOK101 cells, a ccRCC-derived VHL-mutant cell line that transcriptionally resembles ccRCCs with poor prognosis^18,19^, with the correct integration pattern both at the genomic and cDNA level (Fig. 1c,d). No wild-type product was observed by genomic PCR indicating homozygous integration at both *EPAS1* alleles (Fig. 1c). Sanger sequencing verified the integration site (Fig. 1e and Supplementary Fig. 1). mCherry positivity of the clones was confirmed by flow cytometry (Fig. 1f) and confocal microscopy (Fig. 1g).

### Functional validation of endogenous HIF2A reporter systems

**Figure 2:**
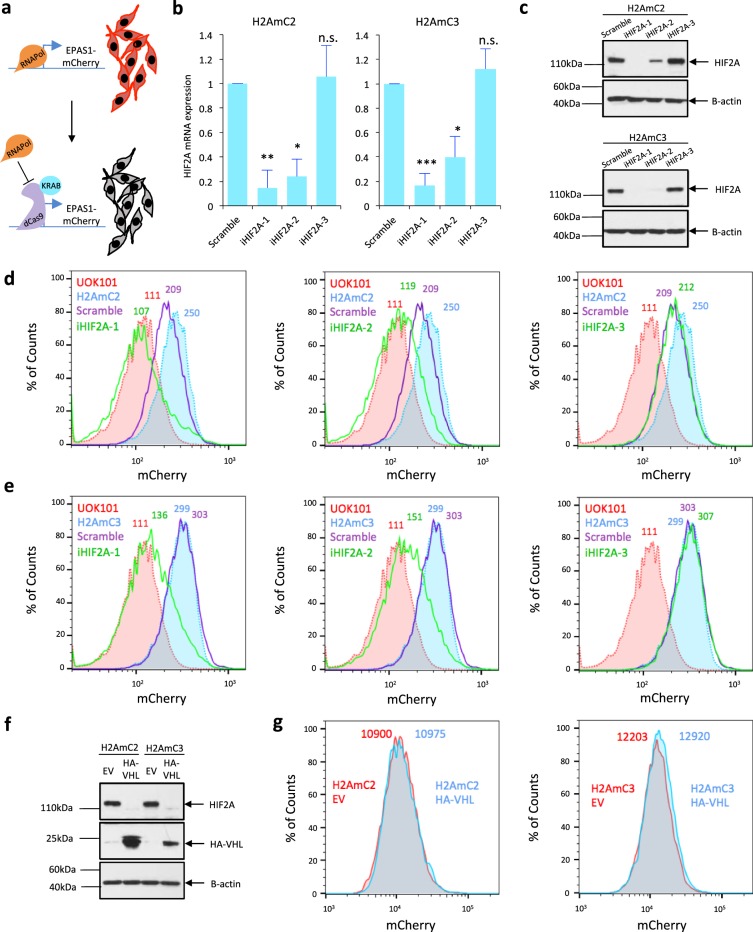
Validation of endogenous HIF2A reporter systems. (**a**) Schematic of CRISPRi-based inhibition of reporter activity. RNAPol, RNA Polymerase II. KRAB, Kruppel associated box. (**b**) Relative HIF2A mRNA levels in H2AmC2 and H2AmC3 dCas9 cells transduced with HIF2A-targeting sgRNAs or a non-targeting control. *P < 0.05, **P < 0.01, ***P < 0.005, n.s., non-significant. P-value by Student’s t-test. (**c**) Western blot analysis of H2AmC2 and H2AmC3 dCas9 cells transduced with HIF2A-targeting sgRNAs or a non-targeting control. B-actin acts as a loading control. Full-length blots are presented in Supplementary Fig. 5. (**d**) FACS analysis of mCherry fluorescence in H2AmC2 cells transduced with different HIF2A-targeting sgRNA constructs. Untransduced H2AmC2 and UOK101 cells serve as a positive and negative control, respectively. (**e**) FACS analysis of mCherry fluorescence in H2AmC3 cells transduced with different HIF2A tandem sgRNAs compared to controls. (**f**) Western blot analysis of VHL-reintroduced H2AmC2 and H2AmC3 cells compared to the empty vector (EV) control. B-actin acts as a loading control. Full-length blots are presented in Supplementary Fig. 5. (**g**) FACS analysis of mCherry fluorescence in H2AmC2 and H2AmC3 cells transduced with HA-VHL and empty vector.

To functionally validate the H2AmC1-3 reporter systems we evaluated mCherry expression alongside HIF2A mRNA and protein expression. We used lentivirally delivered CRISPR interference (CRISPRi)^20^, i.e. sgRNA-mediated recruitment of a repressive dCas9-KRAB protein to a specific genomic locus, to inhibit endogenous HIF2A mRNA expression using three sgRNA constructs (iHIF2A-1, iHIF2A-2, iHIF2A-3) targeting the *EPAS1* transcription start site (Fig. 2a). In comparison to the non-targeting sgRNA control, iHIF2A-1 and iHIF2A-2 caused a significant reduction in HIF2A mRNA level while no reduction was observed in iHIF2A-3 transduced cells (Fig. 2b). These effects translated into similar changes at the protein level (Fig. 2c). The reporter lines expressed a strong HIF2A protein band of the expected size, and the HIF2A antibody did not detect any larger bands of ~150 kDa, the expected size of a HIF2A-mCherry fusion protein, indicating that the T2A peptide was efficiently cleaved in our systems (Supplementary Fig. 2a). Importantly, flow cytometry on the H2AmC2 cells showed that mCherry fluorescence in the non-targeting sgRNA control cells stayed close to the level seen in untransduced cells and cells transduced with iHIF2A-3, while iHIF2A-1 and iHIF2A-2 transduced cells showed a clear reduction in mCherry fluorescence, closely mimicking the parental UOK101 cells (Fig. 2d). These results were replicated in the H2AmC3 cells (Fig. 2e). HIF2A targeting in the H2AmC1 cells resulted in only modest changes in mCherry fluorescence, suggesting the possibility that in this clone mCherry was expressed at least partially independently of HIF2A (data not shown). H2AmC1 was therefore excluded from further analysis. To further test the interdependence of HIF2A mRNA, protein and mCherry fluorescence in our systems we transduced both H2AmC2 and H2AmC3 cells with wildtype *VHL*. As expected, this resulted in a robust reduction in HIF2A protein expression (Fig. 2f). However, no change was observed in HIF2A mRNA expression or mCherry fluorescence (Fig. 2g and Supplementary Fig. 2b,c). These results confirmed that mCherry activity in the H2AmC2 and H2AmC3 systems reflected HIF2A mRNA expression, and they also suggested that HIF2A does not significantly regulate its own expression.

### FACS-based evaluation of reporter activity at the single cell level

**Figure 3:**
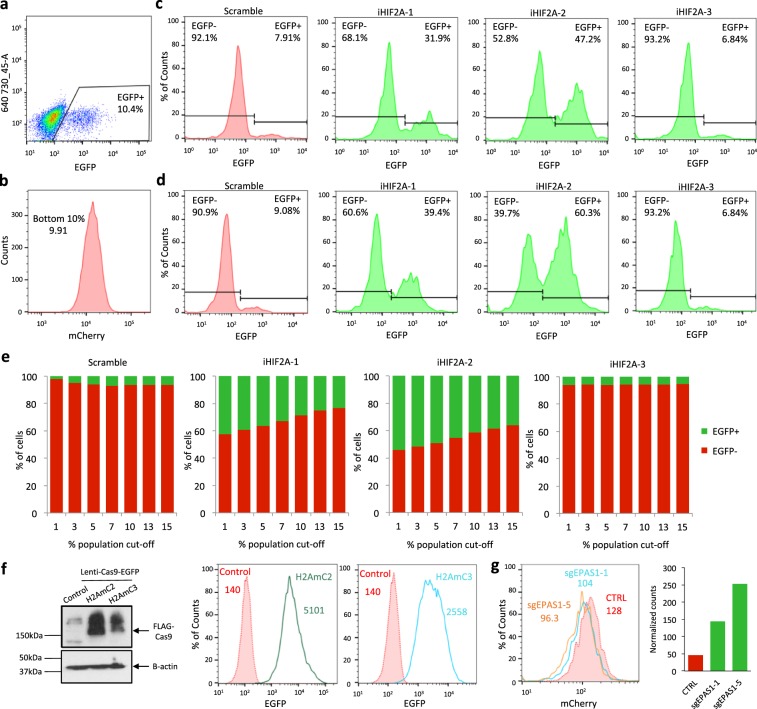
HIF2A sgRNA enrichment as a function of mCherry depletion. (**a**) EGFP fluorescence in sgRNA-transduced H2AmC2 CRISPRi cells. (**b**) mCherry fluorescence of a H2AmC2 population that contains 10% of EGFP+ sgRNA-expressing cells. The bottom 10% of mCherry population was selected for further analysis of EGFP fluorescence. (**c**,**d**) EGFP fluorescence in H2AmC2 (**c**) and H2AmC3 (**d**) cells with the lowest 10% mCherry fluorescence from analysis similar to that shown in panel (**b**). In iHIF2A-1 and iHIF2A-2 cells the fraction of EGFP positive cells is increased compared to the unselected population (panel (a)) or the control or iHIF2A-3 cells, indicating enrichment of HIF2A-targeting sgRNAs in the mCherry low population. (**e**) Relative abundance of EGFP positive (EGFP+) and negative (EGFP-) H2AmC2 cells in populations with different levels of mCherry fluorescence. (**f**) Western blot analysis of H2AmC2 and H2AmC3 cells transduced with lenti-Cas9-EGFP compared to untransduced control cells. B-actin act as a loading control. Full-length blots are presented in Supplementary Fig. 5. Accompanying EGFP fluorescence analysis of single cell derived clones of the respective H2AmC2 and H2AmC3 lenti-Cas9-EGFP pools on the right. (**g**) Left, mCherry fluorescence in H2AmC3-Cas9 cells transduced with non-targeting control sgRNA (CTRL), sgEPAS1-1 or sgEPAS1-5. Right, normalized cell counts for each construct in the combined population with the lowest 10% of mCherry fluorescence.

To test the possibility of using the H2AmC2 and H2AmC3 cells in pooled CRISPR-Cas9-based screening, we transduced them with the HIF2A-targeting sgRNAs under conditions of low multiplicity of infection (MOI) and evaluated the effects on mCherry fluorescence. For these experiments, the sgRNAs were cloned into vectors that also expressed EGFP, which allowed the evaluation of the relationship between the presence of HIF2A-targeting constructs and mCherry activity at the single cell level in a mixed population. Using a population in which ~10% of the cells were EGFP positive (Fig. 3a), leading to an estimated MOI of 0.11 with ~95% of the EGFP positive cells being infected by a single virus, we evaluated the percentage of EGFP positive cells within the population of lowest 10% of mCherry fluorescence (Fig. 3b). As expected, the non-targeting sgRNA or iHIF2A-3 demonstrated no enrichment of EGFP positive cells within the mCherry low population, however, there was a marked increase in EGFP positive cells within the mCherry low fraction in the population that contained 10% of iHIF2A-1 or iHIF2A-2 (Fig. 3c,d). Next, we evaluated the effects of different mCherry population cut-offs on the level of enrichment for EGFP positive cells. This confirmed that decreasing levels of mCherry fluorescence selected for an increasing fraction of EGFP positive cells in iHIF2A-1 and iHIF2A-2 transduced cells whereas no changes were observed in cells transduced with the non-targeting sgRNA or iHIF2A-3 (Fig. 3e and Supplementary Fig. 3a). However, even the lowest 1% of mCherry expressing cells had a significant fraction of EGFP negative cells. Finally, we wanted to test the reporter systems in conjunction with CRISPR-Cas9-based mutational gene targeting. In order to achieve uniform Cas9 expression, we first isolated single cell-derived H2AmC2 and H2AmC3 subclones transduced with a constitutively expressed Cas9-EGFP (Fig. 3f). We then tested the effects of single HIF2A-targeting sgRNAs on mCherry fluorescence at low MOI. As expected, a combined analysis of both control and HIF2A-targeted cells revealed an enrichment of HIF2A-targeted cells in the population of lowest 10% of mCherry fluorescence (Fig. 3g and Supplementary Fig. 3b). In sum, these experiments gave proof-of-principle evidence that a pooled CRISPR-Cas9 genetic screen could be coupled to FACS-based isolation of low mCherry cells in order to identify genes that support HIF2A expression in ccRCC.

### A pooled CRISPR-Cas9 screen for chromatin regulators supporting HIF2A expression

**Figure 4:**
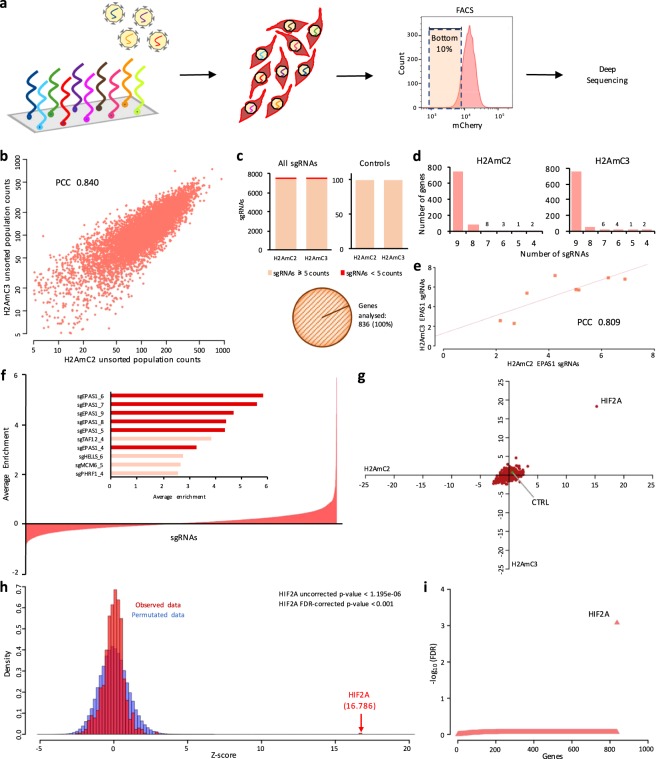
A CRISPR-Cas9 screen targeting chromatin regulators. (**a**) Schematic showing the screening process. A pool of sgRNAs targeting chromatin factors was cloned into a lentiviral vector. H2AmC2 and H2AmC3 clones expressing Cas9 were transduced with the virus, followed by isolation of the cells with the lowest 10% of mCherry fluorescence. sgRNA enrichment was assessed by high-throughput sequencing. (**b**) Correlation plot of the normalized sgRNA counts in unsorted H2AmC2 and H2AmC3 cells. (**c**) Breakdown of total sgRNAs, control sgRNAs and total genes analysed in the screen after the removal of sgRNAs with less than 5 normalized counts in the unsorted population. (**d**) Number of sgRNAs analysed per gene. (**e**) Fold enrichment of HIF2A sgRNAs in H2AmC2 and H2AmC3 cells. (**f**) Average enrichment of all sgRNAs analysed in the screen with a snapshot of the top 10 scoring sgRNAs. (**g**) Z-scores of the median enrichment for all genes in the H2AmC2 and H2AmC3 cells. (**h**) Permutation analysis (1000x) of gene enrichment scores. Observed data in red, simulated data in blue. (**i**) Distribution of FDR-corrected P-values for each gene. PCC, Pearson’s correlation coefficient.

Chromatin factors as supporters of cancer-specific transcriptional programmes are emerging as potential therapeutic target molecules^21^. In order to interrogate the chromatin factor dependencies of HIF2A expression in ccRCC, we generated a focused library of 7617 sgRNAs targeting a total of 836 known and potential chromatin regulators as well as HIF2A as a positive control (Supplementary Tables 1 and 2). The library contained on average nine sgRNAs per gene and 100 non-targeting negative controls. A total of 120 million H2AmC2 and H2AmC3 cells were transduced with the chromatin library at a low MOI, resulting in ~12 million infected cells and thus ensuring >1000X representation of sgRNAs. We then used FACS to isolate the population with the lowest mCherry expression and assessed the representation of sgRNAs by high-throughput sequencing (Fig. 4a). Unsorted cells propagated for an equal time were used as controls. We observed a good correlation between the experimental systems (Fig. 4b and Supplementary Table 2). Only <1.5% of sgRNAs had <5 counts/million reads in the initial population and excluding such sgRNAs retained 100% of the target genes (Fig. 4c) with most genes retaining all sgRNAs (Fig. 4d). The excluded sgRNAs were either lost during library preparation due to technical reasons, or they targeted genes that affected cell viability, such as *SF3B3* and *PCNA*, genes included as control genes in the library. A formal analysis comparing the unsorted control samples to the plasmid that was used for virus production confirmed that known essential genes^22^ were highly represented among the most significantly depleted genes (Supplementary Fig. 4). Looking specifically at HIF2A as a positive control, we found that 8/9 sgRNAs showed evidence of enrichment in the mCherry low population with good correlation between the two reporter systems (Fig. 4e), one sgRNA was excluded due to low representation. In a global analysis, HIF2A sgRNAs stood out as being the most enriched, with 6/10 top scoring sgRNAs targeting HIF2A (Fig. 4f). In a gene level analysis combining data from all sgRNAs for each gene, HIF2A was by far the most strongly enriched gene in the mCherry low population (Fig. 4g). To test the statistical significance of the result we used a permutation-based approach to calculate empirical P-values for the gene level enrichment scores. Comparing the observed data to an expected background clearly highlighted HIF2A as the most significant hit with an adjusted P-value of <0.001 (nominal P-value < 1.195e-06) (Fig. 4h,i). However, no other factor reached statistical significance, a result confirmed by manual inspection of the top-scoring sgRNAs: no other gene contained more than one sgRNA within the top 50 of the list whereas non-targeting control sgRNAs appeared twice. In sum, the only significant hit from our screen was the positive control HIF2A, confirming the technical validity of our approach, but we found no evidence for individual chromatin factors that would be required for the strong HIF2A expression in ccRCC.

## Discussion

We report the development of endogenous reporter systems for HIF2A expression in ccRCC cells and their application in high-throughput functional CRISPR-Cas9-based loss-of-function screening. HIF2A is a critical mediator of ccRCC development and a validated therapeutic target^23–25^. Additionally, varying sensitivity of ccRCC cells to HIF2A inhibition has been attributed to different levels of HIF2A expression as well as acquired mutations in the HIF2A complex^23,25^. Yet, the mechanisms that regulate the high and specific HIF2A expression in ccRCC are incompletely understood. Given the recent evidence that the expression of oncogenic drivers, such as *MYC*, can be dependent on single chromatin modifiers^3^, we explored the chromatin factor dependencies of HIF2A expression using our systems. Despite strong signal for HIF2A, the positive control in the screen, we found little evidence for the existence of individual chromatin factors that would be essential for HIF2A expression in ccRCC. This suggests redundancy within the HIF2A gene regulatory machinery, possibly complicating efforts that aim at targeting HIF2A expression in the therapeutic setting^26^.

CRISPR-Cas9-based genome editing has opened up unprecedented possibilities for biological research. The general efficiency of CRISPR-Cas9 gene editing across the many possible biological contexts remains to be established, however. For example, the degree to which an intact homology-directed repair (HDR) pathway is required for HDR-targeted gene editing in cancer cell lines remains unclear. ccRCCs often carry mutations in the histone methyltransferase *SETD2*, which promotes homologous repair^27–29^. This suggests that defects in some DNA repair pathways may be a requirement for ccRCC development, possibly hindering the efficiency of HDR-mediated gene editing in ccRCC cells. Our results show that at least some ccRCC cell lines are amenable for HDR-based gene editing, but the efficiency appears to be lower than what has been reported in some other systems^14^. Several ccRCC cell lines tested also failed to integrate the fluorescent reporter (data not shown). Alternative strategies that depend on smaller template plasmids and NHEJ-based DNA repair could make gene editing-based development of experimental ccRCC systems more efficient^30^.

While the strong signal for HIF2A in our genetic screen gives technical validation to both the reporter systems and the screening approach, it remains possible that the systems are not sensitive enough to identify factors that only subtly affect HIF2A expression. It is also possible that some chromatin factors were not efficiently targeted due to technical reasons. An alternative interpretation suggests, however, that simultaneous targeting of multiple chromatin factors may be required for efficient inhibition of HIF2A expression. Combinatorial CRISPR-Cas9 screens could thus represent an interesting avenue forward. Targeting alternative gene sets or performing genome-wide screens could also give new insight into HIF2A regulation. The endogenous transcriptional reporters developed herein could also be suitable for unbiased functional analysis of gene regulatory element function^31,32^.

In conclusion, we have developed and validated experimental systems for the identification of factors that support HIF2A expression in ccRCC cells. We demonstrate that these systems are compatible with pooled genetic screens. Combined with microscopy they may also be compatible with small molecule screens in an arrayed format. More generally, similar systems could be useful for the identification of transcriptional dependencies in other cancer contexts as well.

## Methods

### Cells and Reagents

UOK101 cell were obtained from the National Cancer Institute (NCI). The identity of the UOK101 cells has been confirmed by Sanger sequencing-based detection of a previously identified homozygous *VHL* mutation^19,33^ in June 2018, and STR analysis confirmed that the UOK101 cells had not been cross-contaminated with other commonly used cell lines. The cancer cell lines have been confirmed negative for mycoplasma by biannual tests using the MycoAlert™ Mycoplasma Detection Kit (Lonza, LT07-318). UOK101 cells were cultured in RPMI-1640. HEK293T cells, used for lentivirus production, were cultured in DMEM. Both media were supplemented with 10% fetal bovine serum (FBS), penicillin (100 U ml^−1^) and streptomycin (μg ml^−1^). Cell lines were used in experiments within 10 passages from thawing.

### Plasmids and cloning

Px330-U6-Chimeric_BB-CBh-hSpCas9 (Addgene #42230)^34^, pKLV-U6gRNA(BbsI)-PGKPuro2ABFP (Addgene #50946)^35^ and pKLV-U6gRNA(BbsI)-PGKHyg2AEGFP (adapted from Addgene #50946)^35^ were used for sgRNA delivery. The HDR template for targeted gene knock-in was cloned into a truncated pcDNA3.1(−) (Thermo #V79520). The template was generated by step-wise insertion of T2A-mCherry, PCR amplified from pU6-sgCXCR4-2 (Addgene #46917)^20^, PGK-HygR, PCR amplified from pRetroX-Tight-Hygro (Clontech #631034) and the two homology arms that were PCR amplified from genomic DNA by PCR. pHR-SFFV-dCas9-BFP-KRAB (Addgene #46911)^20^ and tandem HIF2A-targeting sgRNA constructs, cloned as previously described^10^, were used for HIF2A CRISPRi. Lenti-Cas9-EGFP (adapted from Addgene #52962)^36^ was used for HIF2A mutagenesis. pLVX-puro (Clontech #632164) was used to express HA-VHL (Addgene #19234)^37^. Primer and sgRNA sequences used in this study are listed in Supplementary Table 3.

### Quantitative RT-PCR

Total RNA from cells was purified using RNAzol RT (Sigma) according to the manufacturer’s protocol. cDNA was synthesized using the High-Capacity cDNA Reverse Transcription Kit (Thermo). qRT-PCR was performed using the StepOnePlus instrument (Thermo) with pre-designed TaqMan gene expression assays (Thermo): EPAS1 (Hs01026149_m1), CXCR4 (Hs00607978_s1), VHL (Hs03046964_s1) and TBP (Hs00427620_m1). Signal was quantified using the double delta Ct method and normalized to TBP as the housekeeping control.

### Western Blotting

Whole-cell extract was used for Western blotting by lysing cells in RIPA buffer (Sigma-Aldrich, Life Science) supplemented with protease inhibitors. Protein was quantified using the PierceTM BCA Protein Assay Kit (ThermoScientific). Primary antibodies for HIF2A (Novus Biologicals NB100-122), FLAG (Sigma #F3165), VHL (BD Biosciences #564183) and β-actin (Sigma #A1978) were used. Secondary antibodies were polyclonal goat anti-mouse immunoglobulins/HRP (Dako) and polyclonal goat anti-rabbit immunoglobulins/HRP (Dako).

### Lentiviral Transduction

Lentivirus was produced by transfecting HEK293T cells with the psPAX2 and pMD2.G viral packaging plasmids using FuGENE 6 transfection reagent (Promega E269A). Viral supernatant was harvested 48 hours after transfection and filtered through a 0.45 mm PVDF syringe filter. Cells were transduced with the virus along with 8 ug/ml of polybrene (Milipore). The number of positive cells was assessed through FACS analysis.

### Fluorescence-Activated Cell Sorting (FACS)

Cells were analysed for fluorescence on an LSR Fortessa (BD Biosciences). Single cells were detected on the basis of FSC-A, FSC-H and SSC-A. mCherry (561 nm/610 nm), EGFP (488 nm/509 nm), and BFP (383 nm/445 nm) fluorescence was measured. Cell sorting was performed on an Influx cell sorter (BD Biosciences) using the same settings as described above. Single cells were sorted onto individual wells of a 96-well plate containing cancer cell-conditioned RPMI-1640 supplemented with 10% fetal bovine serum (FBS), penicillin (100 U ml^−1^) and streptomycin (100 μg ml^−1^). FlowJo software was used to analyse data obtained.

### Immunofluorescence

Cells grown on coverslips in 6-well plates were washed with PBS and incubated with 4% PFA in PBS for 10 min at room temperature (RT). Cells were permeabilized with 0.5% Triton X-100 (Sigma). A drop of ProLong Diamond Antifade Mountant with DAPI (Life Technologies) was placed on microscope slides and coverslips with cells were mounted on them. After 5 min incubation at RT, imaging was done on a Leica TCS SP5 confocal microscope.

### Chromatin Factor sgRNA Library Production

Genes encoding known or putative chromatin factors were identified from previously curated lists^38–41^ (Supplementary Table 1). The library also contained additional control genes, such as known essential genes, as well as 100 non-targeting control sgRNAs^22^. The sequences for the sgRNAs were taken from Wang *et al*.^22^ with nine sequences selected for each gene. Oligos were ordered from Custom Array Inc. Oligos were amplified and cloned into pKLV2-U6gRNA5(BbsI)-PGKpuro2ABFP-W (Addgene #67974)^2^ by Gibson assembly^42^.

### Pooled CRISPR-Cas9 Screening

The lentiviral sgRNA library was produced using HEK293T cells as described above. H2AmC2 and H2AmC3 cells were first transduced with the lentiviral library at varying concentrations to determine the amount needed for a MOI of ~0.11. A total of twelve 15 cm dishes were seeded at 5 million cells each. Twenty-four hours later, i.e. after one doubling time, ~120 million cells were transduced with the lentiviral library at a low MOI, leading to ~10% positive cells and ensuring a >1000x representation of the library. Virus was removed the following day. Puromycin treatment, starting 2 days after transduction, was then applied for a total of 4 days. Three weeks post-transduction, the lowest 10% of the mCherry population from both H2AmC2 and H2AmC3 cells were isolated by FACS and the cells were pelleted along with their respective unsorted controls. DNA was extracted and amplified for the region containing the sgRNAs. Samples were then purified using an Agencourt AMPure XP beads purification protocol. Purified samples were pooled and quantified with Qubit before sending for Illumina sequencing an a HiSeq4000 instrument.

Sequencing results were aligned using Bowtie and normalised to one million reads. Normalised counts of less than five were removed from the analysis. The fold change enrichment was calculated for each sgRNA in both H2AmC2 and H2AmC3 by calculating the fold change of the sorted samples compared to their respective unsorted control sample. Next, the median of all the remaining sgRNAs per gene in each system was calculated. This data was normalised to obtain a z-score for each gene. The average of the normalised median enrichment for each gene was then calculated between the two systems. For each gene, empirical P-values were calculated using a resampling-based method with 1000 permutations and multiple testing correction using FDR.

### Statistical Analysis

Statistical analyses were conducted in R and Microsoft Excel. P-values lower than 0.05 were considered statistically significant. For qRT-PCR three independent experiments are shown, each of which is the average of three technical replicates, unless stated otherwise in the figure legends.

## Electronic supplementary material

Supplementary figures

Supplementary Table 1

Supplementary Table 2

## Data Availability

The TCGA data set was downloaded from the TCGA data portal at https://tcga-data.nci.nih.gov/. The datasets and reagents generated during the current study are available from the corresponding author on reasonable request.
