# Supplementary material for: Endogenous HIF2A reporter systems for high-throughput functional screening
Source: Sci Rep. 2018 Aug 13;8:12063. doi: 10.1038/s41598-018-30499-2 (PMC6089976; doi:10.1038/s41598-018-30499-2)
Supplement: Supplementary file 1 — Supplementary figures [file 41598_2018_30499_MOESM1_ESM.pdf]

**SUPPLEMENTARY INFORMATION**

# Endogenous *HIF2A* reporter systems for high-throughput functional screening

M. Nazhif Zaini[1], Saroor A. Patel[1], Saiful E. Syafruddin[1,2], Paulo Rodrigues[1] & Sakari Vanharanta[1]

1) MRC Cancer Unit, University of Cambridge, Hutchison/MRC Research Centre, Box 197, Biomedical Campus, Cambridge, CB2 0XZ, United Kingdom.
2) UKM Medical Molecular Biology Institute, Universiti Kebangsaan Malaysia, Jalan Yaa'cob Latiff, Bandar Tun Razak, 56000 Cheras, Kuala Lumpur, Malaysia.

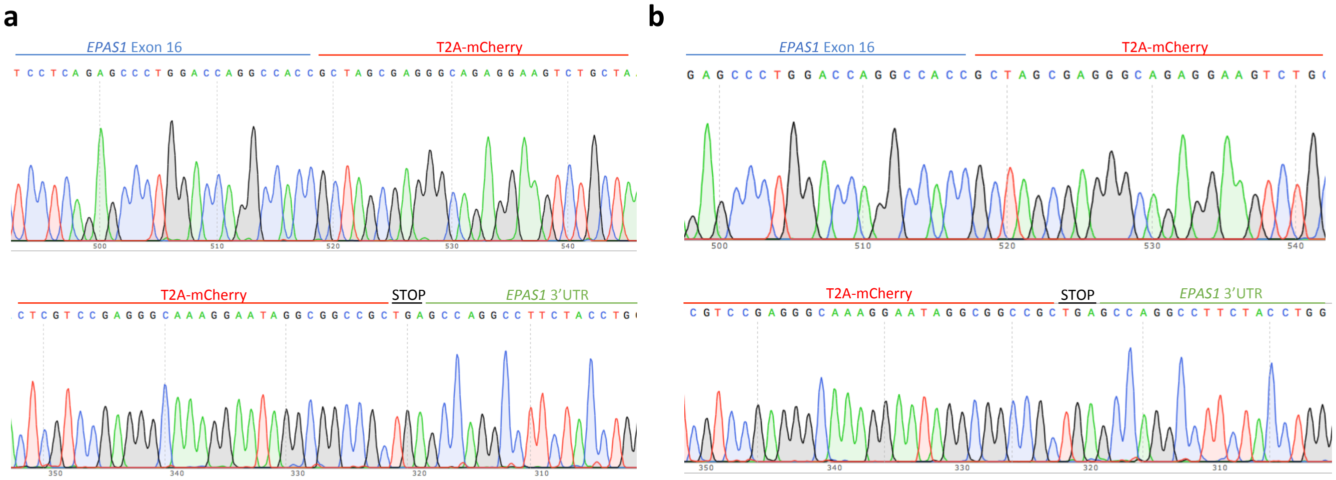


**Supplementary Figure 1. Sequencing of integration sites.** (**a**) Sanger sequencing of the HIF2A-mCherry integration sites in H2AmC3 cells. (**b**) Sanger sequencing of the HIF2A-mCherry integration sites in H2AmC1 cells.

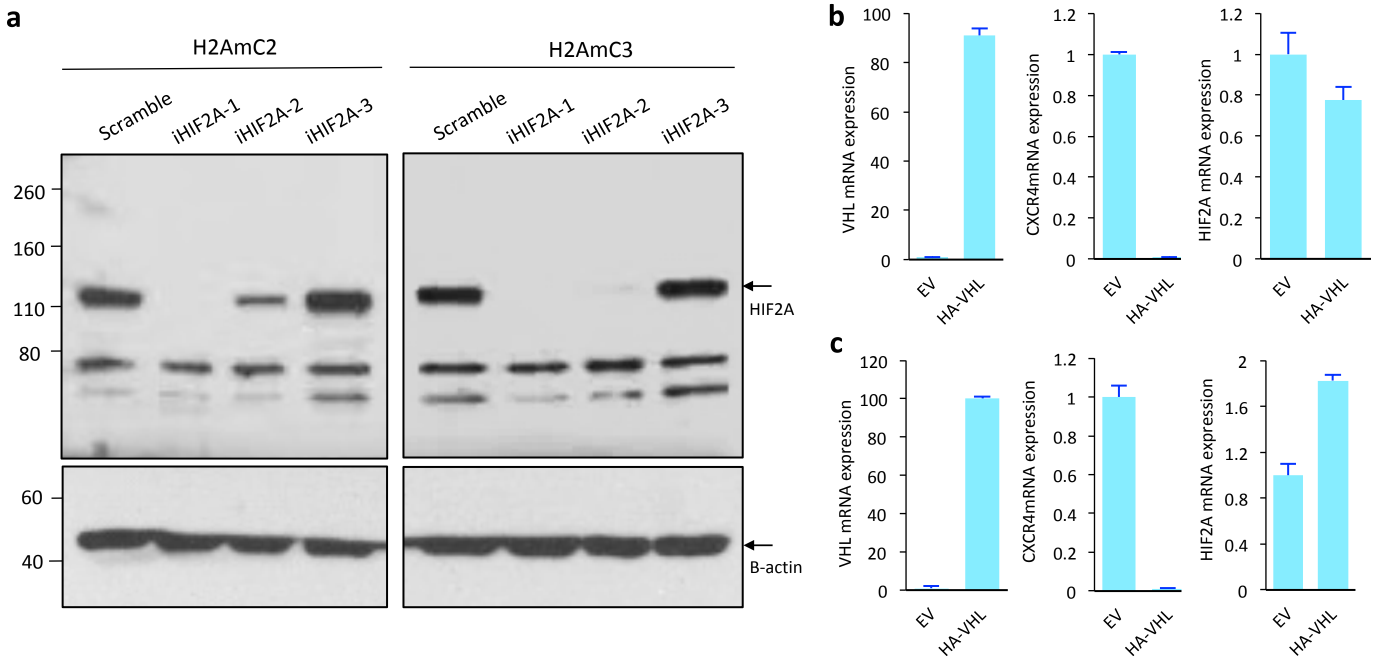


**Supplementary Figure 2. Analysis of HIF2A inhibition.** (**a**) Full Western blot from the analysis of H2AmC2 and H2AmC3 with CRISPRi-based HIF2A targeting shown in Figure 2c. No band detected at ~150kDa, the expected size of an uncleaved HIF2A-T2A-mCherry fusion protein. B-actin used for loading control. Full-length blots are presented in Supplementary Figure 5. (**b**) Relative mRNA levels of VHL, CXCR4 and HIF2A in the H2AmC2 cells transduced with an empty vector (EV) or exogenous VHL (HA-VHL). (**c**) Relative mRNA levels of VHL, CXCR4 and HIF2A in the H2AmC3 cells transduced with an empty vector (EV) or exogenous VHL (HA-VHL).

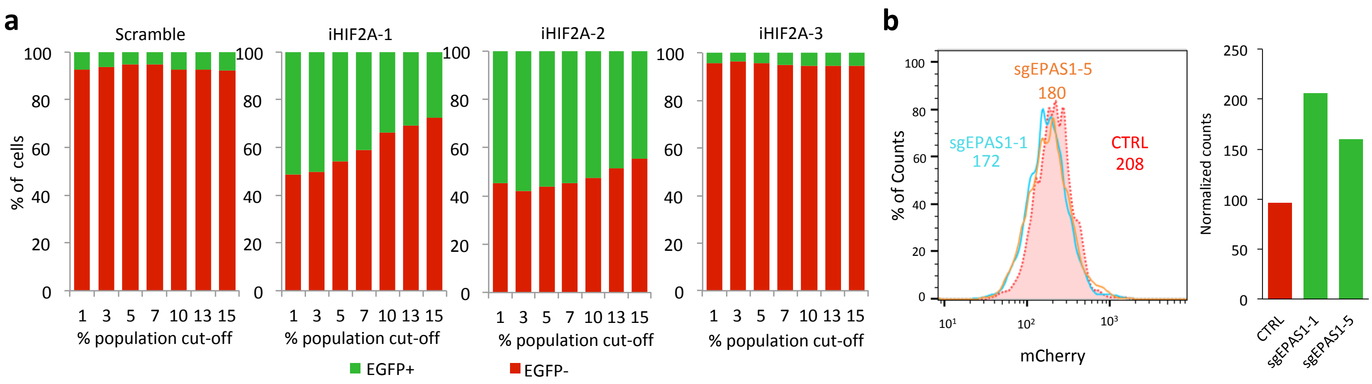


**Supplementary Figure 3. Enrichment analysis of sgRNAs.** (**a**) Relative abundance of EGFP positive (EGFP+) and negative (EGFP-) H2AmC3 cells in populations with different levels of mCherry fluorescence. (**b**) Left, mCherry fluorescence in H2AmC2-Cas9 cells transduced with control (CTRL), sgEPAS1-1 or sgEPAS1-5. Right, normalized cell counts for each construct in the combined population with the lowest 10% of mCherry fluorescence.

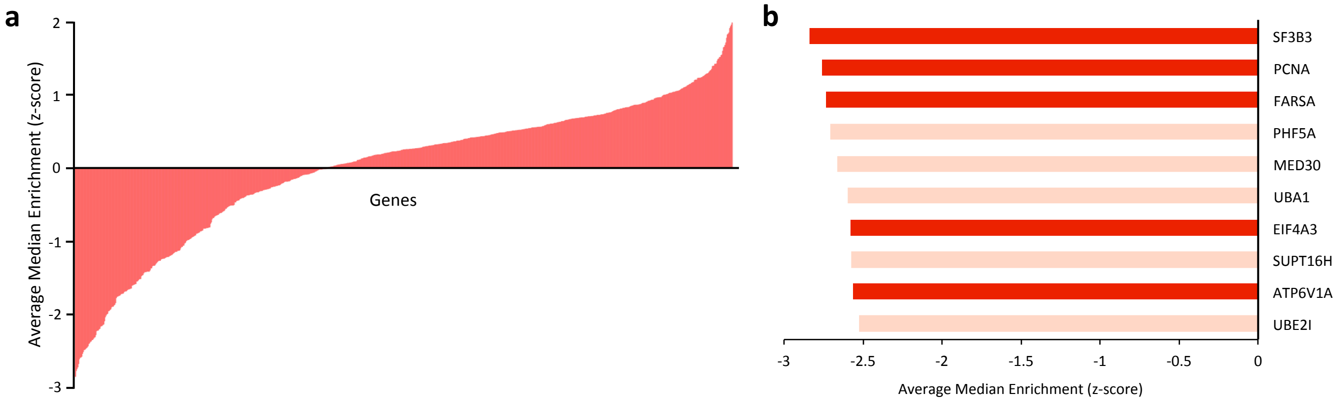


**Supplementary Figure 4. Quality control of CRISPR-Cas9 screen.** (**a**) Average median enrichment for all genes analysed in the screen, comparing the average of the unsorted H2AmC control samples to the plasmid that was used for virus production. (**b**) Top 10 most depleted genes in the unsorted H2AmC control samples when compared to the plasmid. Known essential control genes are highlighted in red.

## Figure 2c and Supplementary Figure 2a

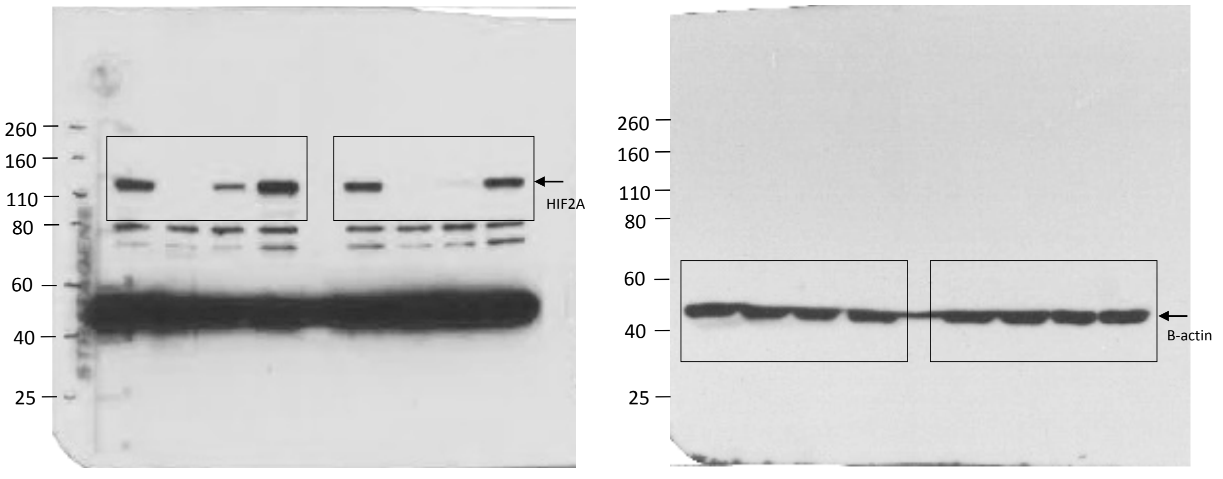


## Figure 2f

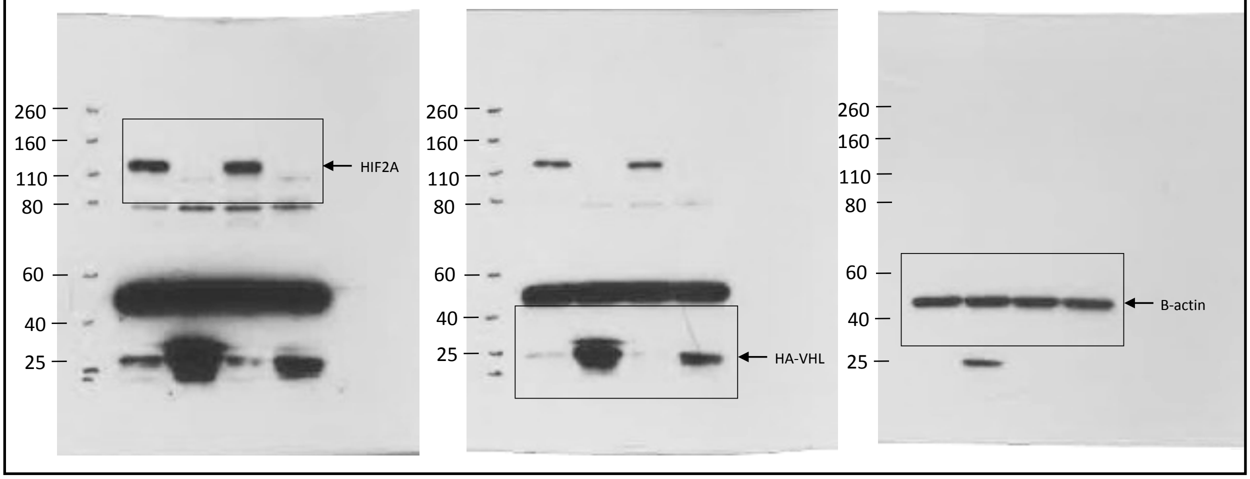


## Figure 3f

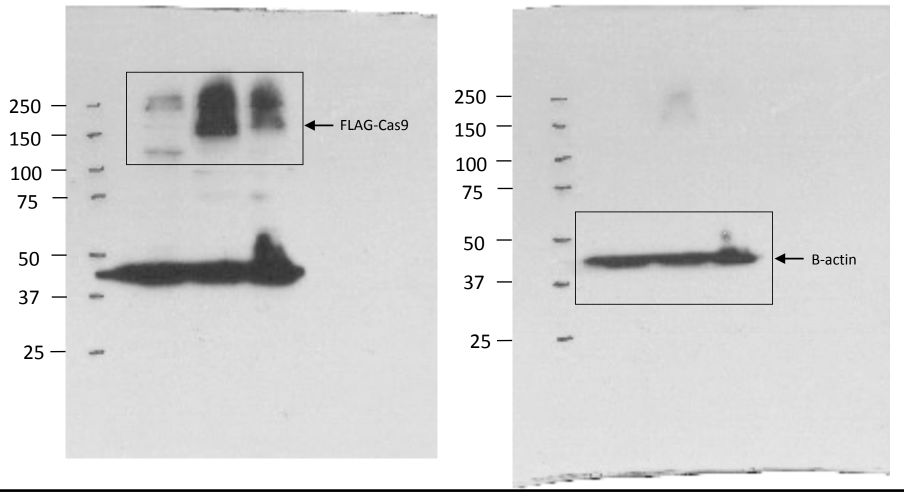


**Supplementary Figure 5.** Full Western blot scans.

| No. | Name | Primer Sequence (5' - 3') |
|---|---|---|
| 1 | EPAS1 Exon 16 sgRNA sequence | TGGACCAGGCCACCTGAGCC |
| 2 | EPAS1 cDNA Sequencing Primer Forward | ATAAGTTCACCCAAAACCCC |
| 3 | EPAS1 cDNA Sequencing Primer Reverse | CCCTCGCCCTCGATCTCGAA |
| 4 | EPAS1 Genomic Sequencing Primer Forward | GGATTTTCAGACTGTTGAAT |
| 5 | EPAS1 Genomic Sequencing Primer Reverse | CCTACAGAAGAACAGACATG |
| 6 | Scramble sgRNA sequence | GAGTGTCGTCGTTGCTCCTA and GGAGATGCATCGAAGTCGAT |
| 7 | iHIF2A-1 sgRNA sequence | TCGCGAGTGTAAAGCTCCCG and ACAGTCTCAGGACACTGCCG |
| 8 | iHIF2A-2 sgRNA sequence | TGGCCCTCGTCCGCTCCCCG |
| 9 | iHIF2A-3 sgRNA sequence | ACAGTCTGGGCTTTTCTCCT and ACCGGGAGCAGGCGAGGGGC |
| 10 | CTRL sgRNA sequence | GAGTGTCGTCGTTGCTCCTA |
| 11 | sgEPAS1-1 sgRNA sequence | GAGTAGCTCGGAGAGGAGGA |
| 12 | sgEPAS1-5 sgRNA sequence | TGAGATTGAGAAGAATGACG |

**Supplementary Table 3.** Primer and sgRNA sequences.
